# A Comprehensive Review of Health-Benefiting Components in Rapeseed Oil

**DOI:** 10.3390/nu15040999

**Published:** 2023-02-16

**Authors:** Junjun Shen, Yejia Liu, Xiaoling Wang, Jie Bai, Lizhong Lin, Feijun Luo, Haiyan Zhong

**Affiliations:** 1 National Engineering Laboratory for Deep Processing of Rice and Byproducts, Central South University of Forestry and Technology, Changsha 410004, China; 2 Faculty of Bioscience and Biotechnology, Central South University of Forestry and Technology, Changsha 410004, China; 3 The Research and Development Department, Hunan Jinjian Cereals Industry, Changde 415001, China; 4 Faculty of Life and Environmental Sciences, Hunan University of Arts and Science, Changde 415006, China

**Keywords:** rapeseed oil, unsaturated fatty acid, functional components, health-benefiting properties

## Abstract

Rapeseed oil is the third most consumed culinary oil in the world. It is well-known for its high content of unsaturated fatty acids, especially polyunsaturated fatty acids, which make it of great nutritional value. There is increasing evidence that a diet rich in unsaturated fatty acids offers health benefits. Although the consumption of rapeseed oil cuts across many areas around the world, the nutritional elements of rapeseed oil and the exact efficacy of the nutrients remain unclear. In this review, we systematically summarized the latest studies on functional rapeseed components to ascertain which component of canola oil contributes to its function. Apart from unsaturated fatty acids, there are nine functional components in rapeseed oil that contribute to its anti-microbial, anti-inflammatory, anti-obesity, anti-diabetic, anti-cancer, neuroprotective, and cardioprotective, among others. These nine functional components are vitamin E, flavonoids, squalene, carotenoids, glucoraphanin, indole-3-Carbinol, sterols, phospholipids, and ferulic acid, which themselves or their derivatives have health-benefiting properties. This review sheds light on the health-benefiting effects of rapeseed oil in the hope of further development of functional foods from rapeseed.

## 1. Introduction

Rapeseed oil is a popular edible oil in China, especially in South China, and the consumption of rapeseed oil in 2019–2020 was up to 27.8 million metric tons [1]. Rapeseed oil is consumed less than palm oil and soybean oil, which ranks third in the world [2]. Bioactive compounds and unsaturated fatty acids (USFAs) are abundant in rapeseed oil and are more than those in animal fat. Saturated fatty acids (SFAs) may be used to evaluate the level of low-density lipoproteins and cholesterol, which are indicators for diagnosing coronary diseases [3,4,5]. On the contrary, USFAs can lower cholesterol levels [2]. Rapeseed oil is also known as canola oil or Brassica oil. Brassica is widespread in various conditions all over the world [6]. It is reported that Canada, Europe, and China are the leading producers of rapeseed oil in the world. In 1979, the Western Canadian oil seed Association proposed the term “canola” to describe the rapeseed varieties that embrace less than 5% erucic acid and less than 40 umol/g of glucosinolates, which is referred to as “double low” rapeseed oil [7]. Although the production is after palm oil and soybean oil, compared with palm oil, the SFA in rapeseed oil is lower than in palm oil which daily intake can lead to cardiovascular and cerebrovascular diseases [8,9]. Meanwhile, compared with soybean oil, rapeseeds have higher oil content.

Rapeseed oil originates from the seeds of brassica plants, which include *Brassica Rapa*, *Brassica napus*. The brassica plant is a significant cash crop because of the high oil content of its seeds [2]. During the year 1975 to 2016, more than 1.9 billion adults were overweight, which can lead to a series of diseases, such as obesity, cardiovascular diseases, hypertension, and hyperlipidemia [3]. Thus, nutritious and digestible foods are urgently needed. Rapeseed oil contains lots of USFAs and bioactive compounds, which makes it beneficial to human health. These components can be classified as antioxidants; however, most rapeseeds have low erucic acid and low glucosinolate content [1]. The nutrients of the phytochemical compound are either water soluble or lipid soluble. This makes lipids of great importance to health. The bioactive compounds in rapeseed consist of phenolic acids, phytosterols, diglycerides, flavones, vitamin E, and flavonols. Both α-linolenic acid and linoleic acid fatty acids are essential fatty acids for humans since they must be consumed from the diet. They cannot be synthesized in the human body due to the lack of specific enzymes [10]. Obviously, the advantage of rapeseed oil is rich in unsaturated fatty acids and especially famous for its high content of oleic acid and linoleic acid. However, the disadvantage of rapeseed oil is a few varieties of rapeseed contain not little erucic acid and glucosinolate which are harmful to people [11,12,13].

Much of the focus on vegetable oils is on the composition of fatty acids previously. There are a lot of functional components in rapeseed and the biological functions of the components are scarcely systematically discussed. In this review, we systematically summarized the functional components of rapeseed oil, and the content and biological functions are clarified in depth. Thus, this review shed a light on developing high-value and nutritious rapeseed oil or extracting functional components from rapeseed for high-value-added functional foods or subsidiary drugs.

## 2. Unsaturated Fatty Acids

It is well-known that USFAs have a lot of health benefits since polyunsaturated fatty acids (PUFAs) cannot be synthesized in the human body and must be obtained from the diet [2]. Many people make their choice of culinary oils based on their nutritional value, and oils containing rich amounts of PUFA are preferred. Rapeseed oil is rich in oleic, linoleic, and γ-linolenic acids. Specifically, many kinds of rapeseed contain 75% oleic acid. All USFAs in rapeseed account for approximately 90% of the total fatty acids composition. Rapeseed oil has a higher proportion of oleic acid than mustard oil and peanut oil; meanwhile, rapeseed oil has a lower proportion of SFAs than soybean oil and sunflower oil. Perrier et al. reported that rapeseed oil is composed of monounsaturated fatty acids (MUFAs), which account for the largest proportion, SFAs, and polyunsaturated fatty acids (PUFAs) [14]. Lewinska et al. found that MUFAs in rapeseed oil account for 62.9% of the total fatty acids, which is the highest content. The content of each component of rapeseed oil is summarized in Table 1 [6,15,16]. Among the PUFAs, oleic acid possesses the highest amount, followed by linoleic and γ-linolenic acids. It is found that people from the Mediterranean region consuming high MUFAs have a low risk of cancers of the skin, breast, and colon, as well as coronary heart diseases [10]. Oleic acid is more heat-stable and oxidation-stable than linoleic acid, and it accounts for 46–66.03% of rapeseed oil [2,6,15,16]. Oleic acid is considered as a phytochemical compound that can ameliorate cardiovascular diseases [17]. Since linoleic acid has one more olefinic bond than oleic acid, the antioxidant effect of linoleic is better than that of oleic acid. Linoleic acid is a nutritional component since it is an essential fatty acid that is important to the human body’s maintenance. Linoleic acid is useful to the human skin’s integrity, immune system, cell membrane, and eicosanoid constitution [2]. Omega-6 fatty acids are famous for their healthcare action, of which linoleic acid and its derivatives, such as γ-linolenic acid, are abundantly constituents in rapeseed oil. It has been revealed that a diet rich in γ-linolenic acid could attenuate high blood lipid, high blood pressure, and skin perspiration [18,19]. γ-linolenic acid also has certain physiological functions, including anti-cancer, anti-thrombotic cardio-cerebrovascular, and anti-diabetic functions, whereas α-linolenic possesses physiological functions, such as anti-atherosclerotic, weight loss, blood lipid-lowering, and cardiovascular and cerebrovascular disease-preventing functions [20,21,22]. The special content of fatty acids in rapeseed oil could lead to a variety of biological functions, which are beneficial for human health.

## 3. Bioactive Compounds

### 3.1. Vitamin E

Vitamin E exists widely in many plants and is abundant in many kinds of plant seeds; it is a fat-soluble vitamin [20]. In plant seeds, vitamin E is found in high concentrations in the seed coat and embryo. In rapeseed oil, it is revealed that the concentration of vitamin E is up to 608.90 mg/Kg (Table 2) [23]. In another study by Xiao et al. the vitamin E content was within the range of 8.3–727.6 mg/Kg (Table 2) [7]. There are eight components (α-, β-, γ-, δ-tocopherol, and α-, β-, γ-, δ-tocotrienol) that form vitamin E. Tocotrienols are similar to tocopherol but each of them has an unsaturated isoprenoid side chain [24,25]. Other forms of vitamin E are less bioavailable in blood and tissues than α-tocopherol [26]. It is easy to take away at the refining step, and it has strong oxidation resistance. It is found that the refining step is most likely to take away the α-tocopherol [27]. In rapeseed oil, tocopherol is more abundant than tocotrienol. Tocopherols can reduce the risk of degenerative diseases that affect the nervous system and muscles [28]. In addition, tocopherols could prevent atherosclerosis and related cardiovascular diseases [29,30]. It is reported that both γ-tocotrienol and δ-tocotrienol have great antioxidant activity, which can inhibit the spoilage of rapeseed oils during storage, thus prolonging the shelf life. Furthermore, tocotrienols have been identified to possess anti-inflammatory and anti-cancer effects [31,32,33,34]. However, the concentration of γ- or δ-tocopherol that can kill half of the cancer cells, thus reaching IC50, would be a much higher concentration (25 μM or 50 μM) [35,36,37]. There are controversies over whether vitamin E can suppress Alzheimer’s disease. It is considered that supplementations with vitamins E and C can prevent cognitive decline [38]. Thus, it is proposed as a treatment for Alzheimer’s disease [39,40].

### 3.2. Flavonoids

Flavonoids are small phenolic molecules that possess a 2-phenyl chromogen ketone parent nucleus. They belong to a diverse class of plant phytochemical metabolites and are abundant in rapeseed [41,42,43]. They are categorized according to the position of the hydroxyl group and exist in the form of a bound flavonoid glycoside or a free flavonoid anhydride [44,45]. In general, flavonoids consist of flavonols, flavan-3-ols, flavanones, isoflavones, and anthocyanidins, among others [46]. They harbor several physiological functions, such as preventing UV, pigmentation, stimulation of nitrogen-fixing nodules, and other health-improving functions [47]. These include the isomers of flavonoids and their hydrogenation and reduced products, most of which exist in the form of glycosides or carbohydrate groups combined with sugars in plants, and a few of them exist in the free form [48]. In rapeseed oil, it has been reported that the total flavonoids are around 164.1 mg/Kg (Table 3) [49]. All flavonoid components are shown in Table 3 [49,50]. In a study by Zeb and Rahman, the component of flavonoids in *Brassica Juncea* L. was evaluated and the results are listed in Table 3 [50].

Quercetin and kaempferol are the most common flavonoids in the human diet and are present as complex glycosides in Brassica species [48,49,51]. Flavonoids act as antioxidants [52] and shielding components [53] in plants and are of special interest due to their antioxidant activity, as well as anti-inflammatory and anti-carcinogenic effects in humans [44,54,55,56,57,58]. Flavonols from copigments with anthocyanins contribute to the seed color, and the oxidation of proanthocyanidins with seed maturation forms oxidized tannins and brown color [56].

Previous studies identified flavonoid glucosides, such as kaempferol-3-sinapoylsophoroside, kaempferol-3-sinapoylsophoroside-glucoside, kaempferol-3-sophoroside, kaempferol-3-sophoroside-7-glucoside, kaempferol-3-sinapoylglucoside-7-sophoroside, and kaempferol-6-sinapoylglucoside-3,7-diglucoside were reported in rapeseed [58,59]. These compounds would fade away by degrees [1]. It has also been shown that flavonoids could ameliorate platelet aggregation, protect against lipid peroxidation, and promote mitochondrial biogenesis [60]. Since quercetin can scavenge free radicals, it has been shown to possess anti-aging effects [61].

Furthermore, a diet rich in quercetin has a variety of health-promoting benefits, such as reduction of inflammation, coagulation, hyperglycemia, and hypertension. In addition, clinical studies have shown that quercetin supplementation is used in the prevention and treatment of various chronic diseases, such as cardiovascular disorders [62]. Kaempferol can significantly attenuate cerebral ischemic reperfusion injury by inducing autophagy in rats via the AMPK/mTOR signal pathway [63].

### 3.3. Squalene

Squalene, as a precursor of other sterols, exists in various vegetable oils. The content differs with the vegetable type, cultivar, agronomic factors, and extraction methods, among others [64,65]. In rapeseed oil, the content of squalene is reported to be approximately 47.8 mg/Kg, which is far less than that in refined olive oil (4784.28 mg/Kg).

The antioxidant activity of squalene is not strong; however, they are difficult to inhibit unless primary antioxidants are present [66,67]. Squalene, a biofunctional lipid compound, is reported to have diverse bioactivities ranging from cardioprotective, antioxidant, chemopreventive, anti-cancerous, anti-lipidemic, and membrane-stabilizing properties, among others [68]. A previous study showed that squalene could enhance serum high-density lipoprotein cholesterol levels and reduce oxidative stress [69]. It has been revealed that squalene and post-squalene metabolites are modulators of hepatic transcriptional changes in rabbits, thus protecting the liver against dysfunction [70]. Mikołajczak et al. reported that squalene in rapeseed oil was 21.8 mg/Kg (Table 4) [71].

### 3.4. Carotenoids

Carotenoids are red, yellow, and orange tetraterpenoid pigments that are universally synthesized by various plants, animals, and microorganisms, especially in rapeseed [72]. In general, the content of lutein in rapeseed oil is higher than that of β-carotene as shown in Table 5. In a previous study, it was found that β-carotene not only exists in cold-press rapeseed oil, but also in rapeseed oil acquired through other processes, such as hot-press, leaching, and aqueous enzymatic extraction [73]. Carotenoids are powerful antioxidants, particularly for neutralizing superoxide anions [74]. It is recognized that carotenoids could inhibit the synthesis of tumor necrotic factor (TNF)-α in monocytes and macrophages and suppress the expression of Toll-like receptors 2 and 4 in human monocytes [75]. Carotenoids play different significant functions in the brain and have several medicinal properties, including neuroplasticity enhancement, antioxidant, anti-inflammatory, anti-diabetes, and anti-apoptotic potentials [76,77]. It also protects against UV damage [78]. It is reported that carotenoids exist in the form of β-carotene and xanthophylls in rapeseed. Lutein (3R,3′R,6′R)-β, ε-carotene-3,3′-diol) is derived from carotenoid -(6′R)-β-carotene, which is one of the few xanthophyll carotenoids that provide desirable health benefits in the macula of the human retina [79,80]. Lutein has antioxidant and anti-inflammatory effects and acts as a yellow filter in humans. Lutein is an extremely strong DNA polymerase inhibitor of polymerase β and λ, but cannot decrease the activities of mammalian polymerase α, γ, δ, and ε [81]. Cold-pressed unrefined rapeseed oil, which contains lutein and other carotenoids, plays an important role in anti-inflammation. Lutein and zeaxanthin have been shown to actively protect against heart disease and eye diseases, including macular degeneration [82].

### 3.5. Glucoraphanin (GRN)

GRN, a member of the glucosinolate family, is a well-known nutritional compound and the precursor of sulforaphane (SFN). Both the content of GRN and SFN in rapeseed oil have anti-cancer effects [88,89]. GRN is found in trace amounts in rapeseed oil, specifically within the range of 0–1.53 μmol/g [90] (see Table 6). SFN, the isothiocyanate derivative of GRN, has attracted much attention in recent years because of its significant health-improving effects [91]. It has been shown that SFN could modulate phase I and phase II detoxifying enzymes, blocking cancer and autoimmune diseases [92]. Previous studies have reported that SFN has cardio-protecting [93] and obesity-preventing effects [94]. SFN can cause cell cycle arrest and apoptosis and can be used to treat stomach cancer since it inhibits *Helicobacter pylori* [95,96]. GRN has anti-inflammatory effects since it suppresses cytokines production [97] and has antibacterial effects against Gram-positive and Gram-negative bacteria [98,99]. The present study evaluated the anti-obesity effect of SFN and GRN in broccoli leaf extract (BLE) in 3T3-L1 adipocytes and ob/ob mice [100].

### 3.6. Indole-3-Carbinol

Indole-3-carbinol (I3C) is an important anti-cancer and chemopreventive phytochemical that acts via further hydrolysis of indol-3-methyl isothiocyanate found in rapeseed [101]. The I3C compound has been revealed to inhibit the proliferation of cancer cells by regulating genes involved in growth, signal transduction, and carcinogenesis. It suppresses the expression of drug resistance-related genes and induces apoptosis [102,103,104,105]. Preliminary clinical trials revealed that I3C could be used to protect against hormone-mediated human cancers [106]. 3′3-diindolylmethane is derived from acid-catalyzed condensation of I3C, which has a biological function [107]. Previously, numerous studies have indicated that 3′3-diindolylmethane can exert anti-cancer effects by triggering apoptosis and inhibiting proliferation. Moreover, it has been shown to prevent the migration and invasion of different types of cancer, including hepatic cell carcinoma [108,109], esophageal cancer [110], breast cancer [111], pancreatic cancer [112], prostate cancer [113,114], and colorectal cancer [115]. I3C exerts its anti-microbial activity by causing excess generation of reactive oxygen species [116]. It has been reported that I3C can protect the neurons in rats suffering from ischemic stroke [117]. It has also been revealed that I3C in leaves is higher compared to the stems of rapeseed varieties [88].

### 3.7. Sterols

Phytosterols occur in many plants and therefore exist in various edible oils. Among all the identified phytosterols, β-sitosterol is the most commonly reported. Other significant phytosterols existing in edible plants include campesterol, brassicasterol, cycloartenol, and stigmasterol [118]. As reported by Mo et al. the specific content of each sterol in rapeseed is shown in Table 7 [119]. Phytosterols have been reported to exhibit anti-inflammatory, anti-microbial, and anti-cancer activities [120,121]. These compounds are structurally similar to cholesterol, which exist in animal cells, whereas sterols exist in plants. It is indicated that phytosterols reduce intestinal cholesterol absorption by regulating several transporters [122,123]. It is controversial that several clinical trials revealed that individuals with high low-density lipoproteins and cardiovascular disease might benefit from phytosterols supplements; however, other studies have suggested that phytosterols did not alleviate the risks of cardiovascular disease [124,125,126]. Nevertheless, phytosterols can reduce serum cholesterol, which makes them useful as a substitute for statins, which have side effects [127,128]. Sterols have been shown to ameliorate the risk of first myocardial infarction [129]. Sterols have both antioxidant [130] and immunomodulatory actions [131]. Sitosterol may prevent obesity-related chronic inflammation by blocking the release of interleukin-6 and TNF-α [132]. It has been reported that phytosterols may serve as a complementary therapy for obesity and diabetes [133]. Moreover, studies have also indicated that β-sitosterol can significantly prevent the development of cancer [134,135].

### 3.8. Phospholipids

In rapeseed, phospholipids are very abundant, and they include glycerolphosphatidic acid and phosphatidylinositol, among others [136]. The content of phospholipid is trace which range from 20–2000 ppm as shown in Table 8. There are differences in the form of phospholipids in rapeseed in terms of different storage conditions, even in the form of hemolysis compounds. In general, phospholipids in rapeseed oil are extracted by physical or chemical extraction. The simple method is to select various organic solvents for multiple extractions [137]. Lecithin is an abundant component in the edible oil refining process. Lecithin is commonly used as a food additive or a drug carrier. Since the composition of lecithin is rich in PUFAs, especially phosphatidylcholine, it has been shown to promote brain function [138,139].

### 3.9. Ferulic Acid (FA)

FA is a health-benefit trace compound found in rapeseed oil, which is a hydroxycinnamic acid. A previous study showed that dietary FA attenuated metabolism syndrome-associated hyperuricemia in rats [141]. It was also reported that FA could ameliorate aflatoxin B1-induced duodenal barrier damage in rats [142]. FA effectively prevents high-fat diet-induced fatty liver disease by activating the PPARα signaling pathway to decrease the accumulation of triacylglycerol in the liver and increase the consumption of energy [143]. FA acid has a cardioprotective effect induced by severe endoplasmic reticulum stress [144]. Moreover, FA has various biological functions, such as anti-microbial, anti-inflammatory, anti-diabetic, anti-cancer, and hepatoprotective actions [145,146]. In addition, FA has been found to exert neuroprotective effects by preventing Aβ-fibril formation and inhibiting free radical production [147]. The content of ferulic acid in rapeseed oil is demonstrated in Table 9.

## 4. The Action of Rapeseed Oil on Metabolic Syndrome, Type II Diabetes, and Obesity

Metabolic syndrome is proposed by WHO and is characterized by insulin resistance, abdominal obesity, hypertension, and hyperlipidemia [148,149]. Comparing cold turnip pressed rapeseed oil (CTPRO) containing 96% of MUFA and PUFA to butter in men with metabolic syndrome, butter significantly lowered LDL cholesterol concentration, but did not lower the overall cholesterol in patients suffering from metabolic syndrome [150]. Furthermore, CTPRO could promote insulin sensitivity and reduce postprandial triglyceride concentrations [151]. It is reported by Baxheinrich et al. that low energy density food with a high intake of MUFA and an α-linolenic 3.5 g daily supplementation could reduce the weight and cardiovascular risk in patients with metabolic syndrome [152]. Diabetes mellitus (DM) is identified by chronic hyperglycemia and impaired carbohydrates, protein, and lipid metabolism due to complete or partial disability of insulin secretion/action [153]. It is indicated by Amiri et al. that canola oil could protect against CVD by elevating apolipoprotein A1 levels in patients with T2DM, especially in females [154]. Most of the epidemiologic data on obesity are based on BMI (in kg/m^2^) and use the ranges of 18.5–24.9 for normality, 25–29.9 for overweight, and ≥30 for obesity [155,156]. It is reported that canola oil intake could decrease hepatic steatosis in obese men due to its intrahepatic lipid content and serum-free acids reduction [157].

In healthy people, on the one hand, compared with sunflower oil and saturated fat, canola oil could lower total cholesterol and low-density lipoprotein cholesterol level in the serum in healthy people [158]. On the other hand, compared with palm oil, rapeseed oil could promote 24 h fat oxidation in healthy young males [159]. Hence, both in healthy and sick people rapeseed oil can improve lipid metabolism, as a result, it is a health-benefiting edible oil.

## 5. Conclusions

Rapeseed is globally known as a huge source of valuable nutrients. A significant advantage of rapeseed oil is that it is rich in unsaturated fatty acids. Thus, rapeseed oil has health-promoting effects on diabetes, metabolic syndrome, and type 2 diabetes. Furthermore, it can promote lipid metabolism in healthy people and patients. Each content of rapeseed has its unique biological functions; thus, it can be inferred that rapeseed oil has a series of biological functions. The rapeseed processing technology should be improved to ensure the good retention of its nutrients. To sum up, in order to preserve the functional components as much as possible the cold-pressed process is proposed. Some of the nutrients are lost during the deodorization process, while some are lost during color removal. In addition, the refining processes may cause the loss of vitamin E, flavonoids, carotenoids, and major phospholipids. Therefore, to maintain the nutrients, high temperature and chemical refining should be replaced by physical refining. The rapeseed oil not only supplies vitamin E directly, but it also includes vitamin E derived from carotenoids. Flavonoids are well-known phytochemicals that can be made for drug and healthcare products, which exert antioxidant, anti-inflammatory, and anti-carcinogenic effects in humans. Phospholipids are often used to improve brain supplements, which are very popularly used for adolescents and children. Thus, the optimization of rapeseed processing for maximum retention of nutrients is very crucial, considering its potential benefits to food processing industries and consumers.

## Figures and Tables

**Table 1:** The fatty acids composition of rape seed oil.

Fatty Acids Composition	Oil Content	Origin	Reference
Palmitic acid (C16:0)	2.21–7.99	Eastern Mediterranean, Europe	[6,10,15]
Stearic acid (C18:0)	1–4.34	Eastern Mediterranean, Europe	[6,10,15]
Oleic acid (C18:1n-9)	46–66.03	Eastern Mediterranean, Europe	[6,10,15]
Gadoleic acid (C20:1)	0–4.74	Eastern Mediterranean, Poland	[6,15]
Linoleic acid (C18:2n-6)	13–40.99	Eastern Mediterranean, Europe	[6,10,15]
Linolenic acid (C18:3n-3)	7.85–14.78	Eastern Mediterranean, Europe	[6,10,15]
Saturated fatty acid (SFA)	6.1–15.8	Eastern Mediterranean, Europe	[6,10,15]
MUFA	62.9, 73.39	Europe	[15,16]
PUFA	20.73, 29.6	Europe	[15,16]
Erucic acid	0.4, 1.93	Eastern Mediterranean, Poland	[10,15]

**Table 2:** Contents of vitamin E in rapeseed oil.

Bioactive Components in RSO	Content (mg/kg)	Reference
Vitamin E	608.90	[23]
	8.3–727.6	[7]

**Table 3:** Contents of flavonoids in rapeseed oil.

Bioactive Components in RSO	Content (mg/kg)	Reference
Flavonoids(total)	164.1	[49]
Quercetin-3-feruloylsophoroside	7.98	[50]
Quercetin-3-glucoside	17.6	[50]
Quercetin-3,7-diglucoside	5.01	[50]
Quercetin-3-rutinoside	2.73	[50]
Kaempferol-3-(caffeoyldiglucoside)-7-rhamnoside	3.76	[50]

**Table 4:** Contents of squalene in rapeseed oil.

Bioactive Components in RSO	Content (mg/kg)	Reference
Squalene	21.8	[71]

**Table 5:** Contents of carotenoids in rapeseed oil.

Bioactive Components in RSO	Content (mg/kg)	Reference
Carotenoid (total)	12.01	[82]
	95	[83]
	29.4–358.7	[84]
β-Carotene	1.88	[82]
	6.0–6.7	[84]
	10.8–13.5	[85]
Lutein	31.6 ± 0.1	[86]
	28–350	[84]
	9.42–163	[87]

**Table 6:** Contents of glucoraphanin in rapeseed oil.

Bioactive Components in RSO	Content (μmol/g)	Reference
Glucoraphanin	0–1.53	[7]

**Table 7:** Contents of sterols in rapeseed oil.

Bioactive Components in RSO	Content (mg/kg)	Reference
β-Sitosterol	3597 ± 596	[129]
Campesterol	1837 ± 10	[129]
Brassicasterol	487.9 ± 6.8	[129]
Stigmasterol	34.0 ± 0.6	[129]
Cycloartenol	87.0 ± 1.8	[129]

**Table 8:** Contents of phospholipid in rapeseed oil.

Bioactive Components in RSO	Content (ppm)	Reference
Phospholipid	20–2000	[140]

**Table 9:** Contents of ferulic acid in rapeseed oil.

Bioactive Components in RSO	Content (μg/100g)	Reference
Ferulic acid	5.42	[71]
